# Systematic Review of Genomic Associations with Blood Pressure and Hypertension in Populations with African-Ancestry

**DOI:** 10.3389/fgene.2021.699445

**Published:** 2021-10-20

**Authors:** S. Singh, J-T. Brandenburg, A. Choudhury, F.X. Gómez-Olivé, M. Ramsay

**Affiliations:** ^1^ Sydney Brenner Institute for Molecular Bioscience (SBIMB), Faculty of Health Sciences, University of the Witwatersrand, Johannesburg, South Africa; ^2^ Division of Human Genetics, School of Pathology, National Health Laboratory Service and Faculty of Health Sciences, University of the Witwatersrand, Johannesburg, South Africa; ^3^ MRC/Wits Rural Public Health and Health Transitions Research Unit (Agincourt), School of Public Health, University of the Witwatersrand, Johannesburg, South Africa

**Keywords:** African-ancestry, blood pressure, genetic association, genome-wide association study, hypertension, systematic review

## Abstract

**Background:** Despite hypertension being highly prevalent in individuals with African-ancestry, they are under-represented in large genome-wide association studies. Inclusion of African participants is essential to better understand genetic associations with blood pressure-related traits in Africans. This systematic review critically evaluates existing studies with African-ancestry participants and identifies knowledge gaps.

**Methods:** We followed the PRISMA protocol, HuGE Review handbook to identify literature on original research, in English, on genetic association studies for blood pressure-related traits (systolic and diastolic blood pressure, pulse and mean-arterial pressure, and hypertension) in populations with African-ancestry (January 2007 to April 2020). A narrative synthesis of the evidence was conducted.

**Results:** Twelve studies with African-ancestry participants met the eligibility criteria, within which 10 studies met the additional genetic association data criteria (i.e., reporting only on African-ancestry participants). Across the five blood pressure-related traits, 26 genome-wide significantly associated SNPs were identified, with six SNPs linked to more than one trait, illustrating pleiotropic effects. Among the SNP associations, 12 had not previously been described in non-African studies.

**Discussion:** The limited number of relevant studies highlights the dearth of genomic association studies on participants with African-ancestry, especially those located within Africa. Variations in study methodology, participant inclusion, adjustment for covariates (e.g., antihypertensive medication) and relatively small sample sizes make comparisons challenging, and have resulted in fewer significant associations, compared to large European studies. Regional variation in the prevalence and associated risk factors of hypertension across Africa makes a compelling argument to develop African cohorts to facilitate large genomic studies, using African-centric arrays. Data harmonisation and comparable study designs, such as described in the H3Africa CHAIR initiative, provide a good example toward achieving this goal.

**Other relevant information:** SS and J-TB were funded by the South African National Research Foundation. MR is a South African Research Chair in Genomics and Bioinformatics of African populations hosted by the University of the Witwatersrand, funded by the Department of Science and Innovation, and administered by the NRF. This review was registered at PROSPERO (registration number: CRD42020179221) and OSF (registration DOI: 10.17605/OSF.IO/QT2HA).

## Introduction

Hypertension (HTN) or high blood pressure is a risk factor for premature deaths and disability worldwide (Forouzanfar et al., 2016; Juma et al., 2016), accounting for 17.9 million deaths in 2018. It is present in approximately 22% of the global population (affecting approximately 1 in 4 men and 1 in 5 women), with the highest prevalence observed in Africa (27%) and lowest in America (average of 18%, where North America < 20% and South America 20–24.9%) (WHO, 2016; 2018).

Over the past decade, the genome-wide association study (GWAS) approach has become the preferred approach to identify genetic factors associated with complex diseases and traits, such as HTN and blood pressure (BP) (Tam et al., 2019). GWAS aims to identify genetic variants and genes associated with the regulation and control of BP. The first case-control studies for HTN and quantitative BP phenotypes were conducted in 2007 as part of the Wellcome Trust Case Control Consortium (Burton et al., 2007) and the Framingham Heart Study (Levy et al., 2007). The GWAS Catalog currently includes 5,152 genetic associations with BP based on 164 studies and 589 associations with HTN based on 76 studies (https://www.ebi.ac.uk/gwas, SNP (single nucleotide polymorphisms) trait *p*-value < 1E-5, accessed April 17, 2020).

The importance of increasing the representation of diverse populations in genomic research, including Africans, has been addressed in many studies (Ramsay et al., 2016; Martin et al., 2018; Popejoy et al., 2018; Bentley et al., 2020). Most GWAS studies have focused on the European ancestry populations (Azam and Azizan, 2018), and studies based on African-ancestry participants comprise mainly African Americans (AAs) (Manolio et al., 2009; Franceschini et al., 2013; Franceschini et al., 2016; Taylor et al., 2016; Liang et al., 2017). The first GWAS for HTN in AAs was conducted by Adeyemo et al. (2009). Larger studies are needed to understand the role of genetic variants and gene-environment interactions on BP and related phenotypes in African populations (Hendry et al., 2018).

This review critically evaluates the literature on GWASs for BP and HTN in participants with African-ancestry, using PubMed, PubMed Central (PMC), Scopus, Web of Science (WoS) and the GWAS Catalog databases.

## Methods

### Protocol and Registration

The systematic review was conducted using: 1) The Preferred Reporting Items for Systematic Reviews and Meta-Analysis (PRISMA) framework (Moher et al., 2009), 2) HuGENET™ (HuGE) Review handbook (Little et al., 2006), and 3) Systematic reviews of genetic association studies (Sagoo et al., 2009). The PRISMA checklist can be found in Supplementary Table S1.

To prevent any duplication of systematic reviews based on the topics addressed in this paper, the Cochrane Library (https://www.cochranelibrary.com), PROSPERO (the international prospective register of systematic review, https://www.crd.york.ac.uk/prospero), OSF (Open Science Framework, https://osf.io) and the Research Registry databases (https://www.researchregistry.com) were used to check for similar reviews. This systematic review was registered at PROSPERO database (registration number: CRD42020179221. Available at: https://www.crd.york.ac.uk/prospero/display_record.php?ID=CRD42020179221). The systematic review was also registered with the OSF as recommended by PROSPERO (registration DOI: 10.17605/OSF.IO/QT2HA, available at: https://osf.io/wg2ty), due to the large turnover time for PROSPERO (∼ 4 months).

### Eligibility Criteria

The eligibility criteria were based on study and report characteristics, outlined in Table S 2 (inclusion of articles were based on GWAS with BP traits and/or HTN with African-ancestries). Studies were excluded if they were: 1) not based on the inclusion criteria (Irrelevant articles, i.e., did not include GWAS, African, Human, BP or HTN); 2) published prior to 2007; 3) not based on whole genome arrays (Targeted or Non-genome-wide arrays); 4) not based on conducting a discovery GWAS within the study or focused on environmental interactions (Replication Study Only, Method Only, GxE Only); 4) focused on the effect of antihypertensive medication treatment (Clinical Study); or 5) not an experimental study (Reviews, Non-Journal Articles).

Additional eligibility criteria were included for genetic association data: 1) Meta-analyses across multiple studies (based on multi-ancestries) were included for only African-ancestry data (studies excluded if they were not separated by ethnicity). 2) Secondary studies (based on the same consortium) were included if the trait was different from that described in the primary study (studies excluded if the trait was previously reported by primary study).

### Database Search Strategy

PubMed, Scopus and WoS were searched for relevant papers and additional papers were found using GWAS Catalog and PMC, published between January 2007 and April 2020 (search date: April 8, 2020). Publication references, from papers that met the eligibility criteria, were further searched to improve the search strategy.

**Pubmed, PMC, Scopus and WoS**: Search criteria are shown in Supplementary Table S3: Database search strategy. The query terms consisted of words related to BP and/or HTN, GWAS, genetic association and African-ancestry. MeSH (Medical Subject Headings) terms were searched and included for each keyword. Boolean operators such as AND/OR/NOT were used to string terms together. The initial PubMed search was without filter restrictions. Whereas the Scopus, WoS and PMC searches were further filtered by inclusion of keywords in the abstract, to get papers that were more relevant (Supplementary Table S3).

**GWAS Catalog:** Papers based on the regular expression “*blood pressure*” and “hypertens*” (trait codes: EFO_0004325, EFO_0000537) were searched in the GWAS database, in the “MAPPED_TRAIT” column. These traits included anything related to BP such as HTN, systolic BP (SBP), diastolic BP (DBP), pulse pressure (PP) and mean-arterial pressure (MAP). The study database was further filtered in “BROAD.ANCESTRAL.CATEGORY” column, using “African*” regular expression, to include only studies with African-ancestry participants. The inclusion criteria for papers within the GWAS catalog can be found at https://www.ebi.ac.uk/gwas/docs/methods/criteria.

### Data Collection

Published studies were identified through database searching, against the selection criteria and screened by titles and abstracts. Full articles were obtained for studies that met the inclusion criteria and where inclusion was uncertain. SS recorded reasons for exclusion (full details in Supplementary Table S4: Inclusion/exclusion criteria of the screened records). Screening of the full-text articles, and selected studies in accordance with the inclusion criteria was conducted by SS and checked by RM, BJ, and CA, with final inclusion by consensus.

### Data Extraction and Evaluation

Data was extracted from the included studies in the form of summary tables. All authors screened the extracted data and ambiguities were resolved through discussion. Data was evaluated by following the guidelines defined by Sagoo et al. (2009) i.e., assessment by: risk of bias in individual studies and across studies case/trait adjustment definition (such as HTN, anti-hypertensive medication (AHM) and covariant adjustment), population stratification, reporting of methods used (sample size of a study population, genotyping method and its reliability/accuracy, validation of results, statistical analyses). This reduced publication bias within this systematic review (avoiding risk of bias caused by selective reporting).

### Summary Measures and Synthesis of Results

The summary of population characteristics and phenotype measures for BP traits for the eligible studies were derived from total population calculations for n and percentage average calculations for mean and SD (Supplementary Table S5). Population characteristics were reported only for African-ancestry. Phenotype measures were reported for BP traits i.e., SBP, DBP, PP and MAP (the effect of continuous variables was measured by beta values) and HTN (the effect of binary variables was measured by odds ratio). BP multi-trait analysis, for the SHet and SHom methods were also reported. The summary of genetic associations was reported for only genome-wide associations (p < 5E-8) (Suggestive associations as per study definition were included in supplementary tables, Supplementary Table S6).

### Additional Analyses

The genetic associations were compared with findings in non-African-ancestry studies reported in the GWAS Catalog (under build 37) and further compared with Evangelou et al. (2018) summary statistics data (currently the largest published study with 757,601 European ancestry individuals), to determine which signals were uniquely identified in studies with African-ancestry populations.

## Results

Few studies were identified on genetic association of BP and HTN in African-ancestry populations, using the GWAS approach. Therefore, a narrative synthesis of the results was conducted, instead of a meta-analysis.

### Selection of Publications for Inclusion

**FIGURE 1:**
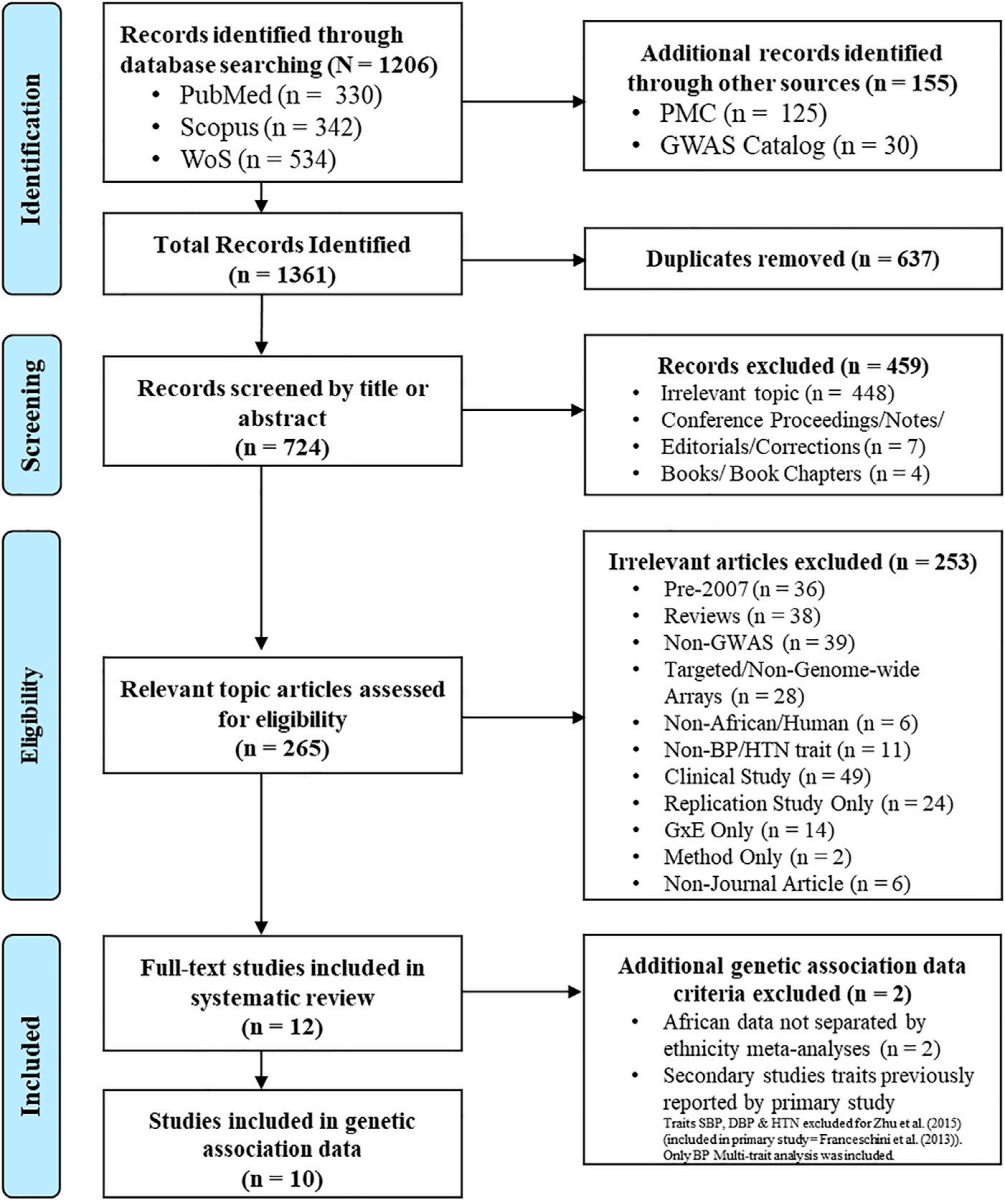
Study schema. Study inclusion/exclusion criteria process, based on PRISMA 2009 flow diagram (Moher et al., 2009). Databases: PubMed (https://pubmed.ncbi.nlm.nih.gov), Scopus (http://www.scopus.com), WoS (http://thomsonreuters.com/thomson-reuters-web-of-science), PMC (https://www.ncbi.nlm.nih.gov/pmc) and GWAS Catalog (https://www.ebi.ac.uk/gwas).

A total of 1,361 papers were identified (PubMed = 330, Scopus = 342, WoS = 534, PMC = 125 and GWAS Catalog = 30, publication references = 0). After removing duplicates, 724 papers remained eligible for screening. After screening by title, abstract and full-text, in cases where it was not clear if inclusion criteria had been met, only 12 papers were considered eligible and retained for the systematic review (Figure 1).

The first GWAS with BP and HTN in an African-ancestry population was conducted in 2009 (Adeyemo et al., 2009), with the most recent being conducted in 2020 lines (Willems et al., 2020). Over the years, the number of publications increased (Supplementary Figure S1).

### Cohort Characteristics

The African-ancestry population size ranged between 815 (Lule et al., 2019) to 31968 (Liang et al., 2017) individuals (Table 1). Most studies were conducted on AAs (study setting = United States for nine studies) (Table 1). Four studies were from participants living in Africa i.e., Nigeria (Franceschini et al., 2013; Zhu et al., 2015) and Uganda (Gurdasani et al., 2019; Lule et al., 2019). All studies, where age was reported, included only adults (> 18 years), with the exception of Lule et al. (2019), which included 10–11 year old Ugandan adolescents. Meta-analyses were conducted for most studies (Supplementary Table S5), with exception of two (Adeyemo et al., 2009; Lule et al., 2019). Most studies included more women (Supplementary Table S5), with exception of Giri et al. (2019) and Lule et al. (2019) which had 8.5 and 48.7% women respectively.

**TABLE 1:** Study characteristics of the 12 eligible publications.

**Publication Year**	**Database pull**	**Age group (Years)**	**Study setting/region**	**Initial size (N)**	Male composition (%)	**Composition**	**African-ancestry size (n)**	**African-ancestry Composition**	**Ethnicity group**	**Africans ancestry only** (%)	**Meta-analysis**	**Consortium**	**References**
2009	GWAS Catalog; PM; PMC; Scopus; WoS	NA	United States	1,017	41.2	509 AA cases, 508 AA controls	1,017	509 AA cases, 508 AA controls	AA	100	No	HUFS	Adeyemo et al. (2009)
2011	GWAS Catalog; PM; PMC; Scopus; WoS	>18	United States	7473	39.7	7,473 AA	7473	7,473 AA	AA	100	Yes	CARe (1a)	Fox et al. (2011)
2013	GWAS Catalog; PM; PMC; Scopus; WoS	≥ 20	United States; Nigera	29378	27.1	28,190 AA, 1,188 Nigerian	29378	28,190 AA, 1,188 Nigerian	AA; African-Ancestry (Nigerian)	100	Yes	COGENT (2a)	Franceschini et al. (2013)
2019	GWAS Catalog; PM	≥ 18	NR; United Kingdom	4,59,777	91.5	MVP (N = 318,891): whites (N = 5,265), blacks (N = 4,671), Asians (N = 3,936) and Hispanics (N = 1,838); United Kingdom Biobank: 140,886 empirically classified white	4671	4,671 Blacks	African-ancestry	1	Yes	^#^MVP and UKB	Giri et al. (2019)
2019	GWAS Catalog; WoS	>18	Uganda, Africa	14126	—	14,126 African-ancestry	14126	14,126 African-ancestry	African	100	Yes	UGR	Gurdasani et al. (2019)
2017	GWAS Catalog; Scopus; WoS	> 60.9	United States	99785	40.5	Non-Hispanic whites (81%; 80,792), Latinos (8%; 8,231), East Asians (7%; 7,243), AAs (3%; 3,058), and South Asians (1%; 461)	3058	3,058 AA	AA	3	Yes	^*^GERA	Hoffmann et al. (2017)
2011	GWAS Catalog; PM; PMC; Scopus; WoS	>18	United States	7917	39.7	4,069 AA cases, 3,848 AA controls; CARe; ARIC (N = 3,269), CARDIA (N = 1,209), CFS (N = 704), JHS (N = 2,200), and MESA (N = 1,737)	7917	4,069 AA cases, 3,848 AA controls	AA	100	Yes	CARe (1b)	Lettre et al. (2011)
2017	GWAS Catalog; PM; PMC; Scopus; WoS	NA	United States; NR	31968	30.3	31,968 of African-ancestry from 19 AA Cohorts and 2 African cohorts	31968	31,968 African-ancestry from 19 AA Cohorts and 2 African cohorts	African ancestry	100	Yes	21 Previous GWASs	Liang et al. (2017)
2019	GWAS Catalog; PM; PMC; Scopus; WoS	10 to 11	Uganda, Africa	815	51.1	815 African-ancestry early adolescents	815	815 African-ancestry early adolescents	African	100	No	EMaBS	Lule et al. (2019)
2020	GWAS Catalog	NA	United States	1,520	39.1	281 AA or Afro-Caribbean, 598 Hispanic or Latin American, 125 East Asian, 516 Europeanls	281	281 AA	AA	18	Yes	#GENNID	Willems et al. (2020)
2019	GWAS Catalog	NA	United States	49839	—	49,839 non-European; 11,863 AA cases, 8,663 Hispanic/Latino cases, 3,102 Asian ancestry cases, 2,645 Native Hawaiian ancestry cases, 342 Native American ancestry cases, 508 cases, 5,289 AA controls, 13,273 Hispanic/Latino controls, 1,561 Asian ancestry controls, 1,060 Native Hawaiian ancestry controls, 293 Native American ancestry controls, 542 controls	17152	11,863 AA cases,5,289AA controls	AA	34	Yes	*PAGE	Wojcik et al. (2019)
2015	PM; PMC; Scopus; WoS	≥ 20	United States; Nigera	29378	—	28,190 AA, 1,188 Nigerian 3000 total used	29378	28,190 AA, 1,188 Nigerian; 3000 total used	AA; African Ancestry (Nigerian)	100	Yes	COGENT (2b)	Zhu et al. (2015)

Paper and population Characteristics of the 12 eligible publications. *Only African ancestry reported in this review (100% African ancestry), #Not 100% African ancestry. (a) Primary study (b) Secondary study. Red = Studies excluded when reporting genetic associations. Cohorts: AADM (Africa America Diabetes Mellitus Study), ARIC (Atherosclerosis Risk in Communities), CARDIA (Coronary Artery Risk Development in Young Adults), CARe (Candidate Gene Association Resource), CFS (Cleveland Family Study), CHS (Cardiovascular Health Study), COGENT (Continental Origins and Genetic Epidemiology Network), DCS (Diabetes Case-control Study), DDS (Durban Diabetes Study), EMaBS (Entebbe Mother and Baby Study), GENNID (GENetics of Noninsulin dependent Diabetes Mellitus), GERA (Genetic Epidemiology Research on Adult Health and Aging), GPC (General Population Cohort), HRC (Haplotype Reference Consortium), HUFS (Howard University Family Study), JHS (Jackson Heart Study).

Eight studies were based on only participants of African-ancestry (Table 1). Of the 4 studies with other ethnicities (Table 1), Wojcik et al. (2019) and Hoffmann et al. (2017) reported ethnicity based GWASs and therefore only the African-ancestry data was reported for this systematic review (34 and 14% African-ancestry, respectively). Giri et al. (2019) and Willems et al. (2020) reported low percentages of African-ancestry within the conducted GWASs i.e., 14% and 18% respectively and the GWAS was based on multiple ethnicities, therefore, these studies were excluded when reporting genetic associations. There were two secondary studies. The Lettre et al. (2011) publication was the secondary study to that reported in Fox et al. (2011), based on the Candidate Gene Association Resource (CARe) consortium. The Zhu et al. (2015) publication was the secondary study of the Franceschini et al. (2013) study and was based on the Continental Origins and Genetic Epidemiology Network (COGENT) consortium.

### Phenotype Measurement

From the studies that reported phenotype characteristics (Table 2), three used the automated Omron device and one used a sphygmomanometer to measure BP. From the reported readings, five studies used the average of the second and third recorded readings as the final reading, with the measurements taken with the patient in a seated position. All BP traits were derived from standard calculation, as reported by respective studies. For five of the studies, if a patient was taking AHM, then SBP and DBP was adjusted by adding 15 andand 10 mmHg (three studies) or adding 10 andand 5 mmHg (two studies), respectively. All publications examined SBP and DBP, with the exception of Lettre et al. (2011) who only looked at HTN as a trait (Supplementary Figure S1). SBP and DBP average measures were reported by most studies (Table 2).

**TABLE 2:** Phenotype characteristics (BP Traits and HTN) of the 12 eligible publications.

**Instrument**	**Reading taken**	**Final reading**	**Patient position**	**Guidelines**	**DNA extracted from**	**BP traits studied**	HTN cases (%)	**AHM (%)**	**SBP (Mean)**	**SBP (SD)**	**DBP (Mean)**	**DBP (SD)**	**PP (Mean)**	**PP (SD)**	**References**
Oscillometric device (Omron)	3X (every 10 min)	Average of 2nd and 3rd	Seated position	JNC7	NA	HTN SBP; DBP	NA	NA	131.3	16.5	81.35	10.95	—	—	Adeyemo et al. (2009)
Random-zero, Mercury and Autimated oscillometric Sphygmomanometer	ARIC and JHS = 3X; CARDIA = After 5 min rest period, 3X (every 1 min); CFS = 9X (3 every 18 h); MESA = 3X (every 1 min)	Average of 2nd and 3rd	Seated position	AHM adjusted = 10 and 5 mmHg added for SBP and DBP	NA	SBP; DBP	—	42.2	125.9	18.6	77.5	11.1	—	—	Fox et al. (2011)
NA	NA	NA	NA	AHM adjusted = 10 and 5 mmHg added for SBP and DBP; Outliers excluded = >4 SDs from mean	NA	HTN; SBP; DBP	26.2	44.9	130.6	20.5	78.1	11.8	—	—	Franceschini et al. (2013)
NA	used earliest recorded measured SBP and DBP	Median SBP and DBP	NA	AHM adjusted = 15 and 10 mmHg added for SBP and DBP; as per Newton-Cheh C et al.2009 and Taylor et al. 2016	Blood	SBP; DBP; PP	—	0.3	136.4	16.0	82.6	11.6	54.0	12.4	Giri et al. (2019)
Automated Omron M6-I	3X (every 3–5 min)	Average of 2nd and 3rd	Seated position	As described previously (Asiki et al., 2013); Fasting 30 min prior to the measurement	Blood	SBP; DBP	—	—	122.8	17	74	10.5	—	—	Gurdasani et al. (2019)
NA	NA	Average of repeated BP measures over a 5-years timespan	NA	AHM adjusted = 15 and 10 mmHg added for SBP and DBP; As per Tobin et al. 2005	NA	SBP; DBP; PP	—	—	127.8	14.8	76.2	9.6	52.1	11.7	Hoffmann et al. (2017)
NA	NA	NA	NA	NA	NA	HTN	31.9	—	—	—	—	—	—	—	Lettre et al. (2011)
NA	NA	NA	NA	AHM adjusted = 15 and 10 mmHg added for SBP and DBP; JNC	NA	HTN; SBP; DBP; BP (multi-trait analysis)	55.5	39.5	133.1	22.5	80.2	13.1	53.0	15.5	Liang et al. (2017)
Automated Omron (M6, HEM‐700) machines	After 5 min rest period, BP measured 3X (every 5 min)	Average of 2nd and 3rd	Seated position	As decribed by Lule et al., 2019; “Pre-hypertension” was defined as systolic or diastolic BP ≥ 90th but <95th percentile	Red cell pellets	SBP; DBP	—	—	106	—	65.3	—	—	—	Lule et al. (2019)
NA	NA	NA	NA	NA	Blood	SBP; DBP	—	—	—	—	—	—	—	—	Willems et al. (2020)
NA	NA	NA	NA	NA	NA	HTN; SBP; DBP	69.16	—	139.66	21.96	85.95	13.43	—	—	Wojcik et al. (2019)
NA	NA	NA	NA	NA	NA	HTN; SBP; DBP; BP (multi-trait analysis)	—	—	—	—	—	—	—	—	Zhu et al. (2015)

### Genotyping and Genome-Wide Association Study Parameters

DNA was extracted from blood samples (Giri et al., 2019; Gurdasani et al., 2019), red cell pellets (Lule et al., 2019) and Epstein-Barr virus (EBV) transformed blood lymphocyte cell lines (Willems et al., 2020) (Table 2).

Genotyping were performed using Illumina arrays (four studies), and Affymetrix arrays (eight studies) (Table 3). While the three initial studies were based on 600 K SNP arrays, studies that are more recent used the 1 M (Affymetrix array studies) and 2.5 M (Illumina studies) arrays. All the studies used standard GWAS quality control (QC) parameters. SNP imputation was done for all studies, with the exception of Adeyemo et al. (2009). A range of reference panels was used, with the most common being the 1,000 Genomes Project reference panel (six studies). Merged reference panels were used for six studies. For example, the African Genome Variation Project (AGVP) reference panel was used by both Ugandan studies (Gurdasani et al., 2019; Lule et al., 2019). The final number of SNPs ranged between 808,465 and 30,072,738 SNPs. Population structure was adjusted for in most studies.

**TABLE 3:** Genotype and analyses characteristics of the 12 eligible publications.

**Array type**	**Array name**	**QC**	**Population stucture/PCA**	**Imputed**	**References Panel**	**Final SNPs number**	**Association**	**Covariates/Confounding variables adjusted for**	**Suggestive sig.**	**Replication**	**References**
GW genotyping array	Affymetrix ® GW Human SNP Array 6.0	Yes	Structure = Non parametric clustering of genotypes using the AWClust algorithm; PC = using eigenstrat method	No	NA	8,08,465	LoRM under an additive model for HTN (PLINK V1.04); LRM for SBP and DBP (PLINK V1.04)	Age, Sex, BMI, First 2 PCs of the genotypes	p < 1E-4	Yes	Adeyemo et al. (2009)
GW genotyping array	Affymetrix GW Human SNP Array 6.0 array	Yes	Structure = MDS; PCs were calculated for all unrelated individuals and predicted forrelated individuals	Yes	Merged: YRI and CEU HapMap-phased haplotypes	2,500,000	LRM under an additive model	Age, Age2, Sex, BMI, First 10 PCs	p < 1E−6	Yes	Fox et al. (2011)
GW genotyping array	Affymetrix GeneChip SNP Array 6.0	Yes	PCA	Yes	Merged: HapMap Phase III and the Human Genome Diversity Project	∼2,420,000	LRM/LMM for family data under additive model	Age2, Sex, BMI, First 10 PCs	p < 1E−5	Yes	Franceschini et al. (2013)
GW genotyping array	Affymetrix Axiom Biobank array designed specifically for the MVP	Yes	PCA using FlashPCA; Clusted by race using K-means approach K1-4	Yes	1000G phase 3, V5	NR	LRM with additive models (untransformed), using SNPTEST-v2.5.4-beta	Age, Age2, Sex, BMI, First 10 PCs	p < 1E−6	Yes	Giri et al. (2019)
GW genotyping array, GW sequencing	Illumina HumanOmni 2.5M BeadChip array(UGR); Illumina HumanOmni Multi-Ethnic GWAS/Exome Array (MEGA pre-commercial v1) using the Infinium Assay a.k.a MEGA Array (DDS and DCC and some of AADM); Affymetrix Axiom GW PanAFR Array Set (AADM)	Yes	PCA; fineSTRUCTURE analysis (Lawson et al., 2012)	Yes	Merged: Uganda Genome Resource, AGVP, 1000G phase 3	>24,423,923	LMM with two random effects	Age, Age2, Sex	None^a^	No	Gurdasani et al. (2019)
GW genotyping array	Affymetrix Axiom array	Yes	PCA; LMM using estimated kinship matrices with leave one chromosome out (LOCO) to account for population substructure and cryptic relatedness with Bolt-LMM	Yes	1000G phase I	>2,696,785	LMM using estimated kinship matrices in Bolt-LMM	Age, Age2, Sex, BMI, First 2 ancestry PCs	p ≤ 1E-2	Yes	Hoffmann et al. (2017)
GW genotyping array	Affymetrix GW Human SNP Array 6.0 platform	Yes	Family structure modeled using LME models; PCA using EIGENSTRAT	Yes	Merged: HapMap phase 2 CEU and YRI data	∼2,740,000	LoRM under an additive genetic model In PLINK	First 10 PCs	p < 1E−6	Yes	Lettre et al. (2011)
GW genotyping array	Either Affymetrix or Illumina	Yes	PCA	Yes	1000G Phase 1	>30,072,738	LRM for unrelated data or by the generalized LMM for family data, under an additive genetic model	Age, Age2, Sex, BMI, up to first 10 PCs	p < 1E−6	Yes	Liang et al. (2017)
GW genotyping array	Illumina HumanOmni2.5M-8 (‘octo’) Beadchip arrays, version 1.1 (Illumina Inc., San Diego, United States)	Yes	Structure = LMM (GCTA V1.22)	Yes	Merged: 1000G, AGVP and UG2G	20,074,711	LMM regression (accounting for population substructure) (GCTA V1.22)	Age, BMI	p < 1E−6	Yes	Lule et al. (2019)
GW genotyping array	Infinium Multi‐Ethnic Global BeadChip (v1.0)	Yes	NA; TransMeta, follows a hierarchical framework that incorporates a kernel matrix K into the covariance structure of the effect size estimates	Yes	EA: HRC; AA, MA & JA: 1000G Phase 3	3,153,931	LMM using GCTA (Log & rank‐based inverse normal transformations)	Age, Sex & self‐reported diabetes status (FEs)	p < 1E–6	No	Willems et al. (2020)
GW genotyping array	MEGA array	Yes	PCA, (SNPRelete in R)	Yes	1000G phase 3	28,263,875	LoRm and LMM, in both SUGEN and GENESIS program	AHM adjustment, (for BP traits only, not HTN), First 10 PCs	None^b^	Yes	Wojcik et al. (2019)
GW genotyping array	Either with Affymetrix or the Illumina whole-genome SNP genotyping arrays	Yes	NA	Yes	NA	∼2,420,000	LRM, additive model; Plink	None	*p* < 5 × 10^–7^	No	Zhu et al. (2015)

GWAS sig. = p < 5 × 10^–8^ for all studies, except for ^a^GWAS sig. = < 5E–9) ^b^GWAS sig. = < 3E–9 for MAF <5%.

For genome-wide association the following tests were performed; 1) Linear regression models (LRMs)/linear mixed models (LMMs) regression, under an additive model, to measure continuous BP traits (i.e., SBP, DBP and PP) and 2) logistic regression models (LoRMs), under an additive model, to measure binary traits (i.e., HTN). All studies adjusted for covariates or confounding variables, with the exception of Zhu et al. (2015). The most common covariates adjusted for in the association testing were age, age2, sex, body mass index (BMI), and first 10 principal components (PCs) of the genotypes. The distributions of association were displayed by means of Manhattan and Q–Q plots for all studies, with most studies (seven) using locus/regional plots. Almost all studies used the standard cutoff for genome-wide significance (p < 5E-8), with exception to Gurdasani et al. (2019) who used p < 5E-9 and Wojcik et al. (2019) who used MAF (minor allele frequency) specific *p*-value thresholds (p < 5E-8 for MAF >5%; p < 3E-9 for MAF <5%). No suggestive significance was reported in these two studies. Replication of the genome-wide associations was conducted for most studies, with three exceptions (Zhu et al., 2015; Gurdasani et al., 2019; Willems et al., 2020).

### Summary of Genetic Associations Detected

**FIGURE 2:**
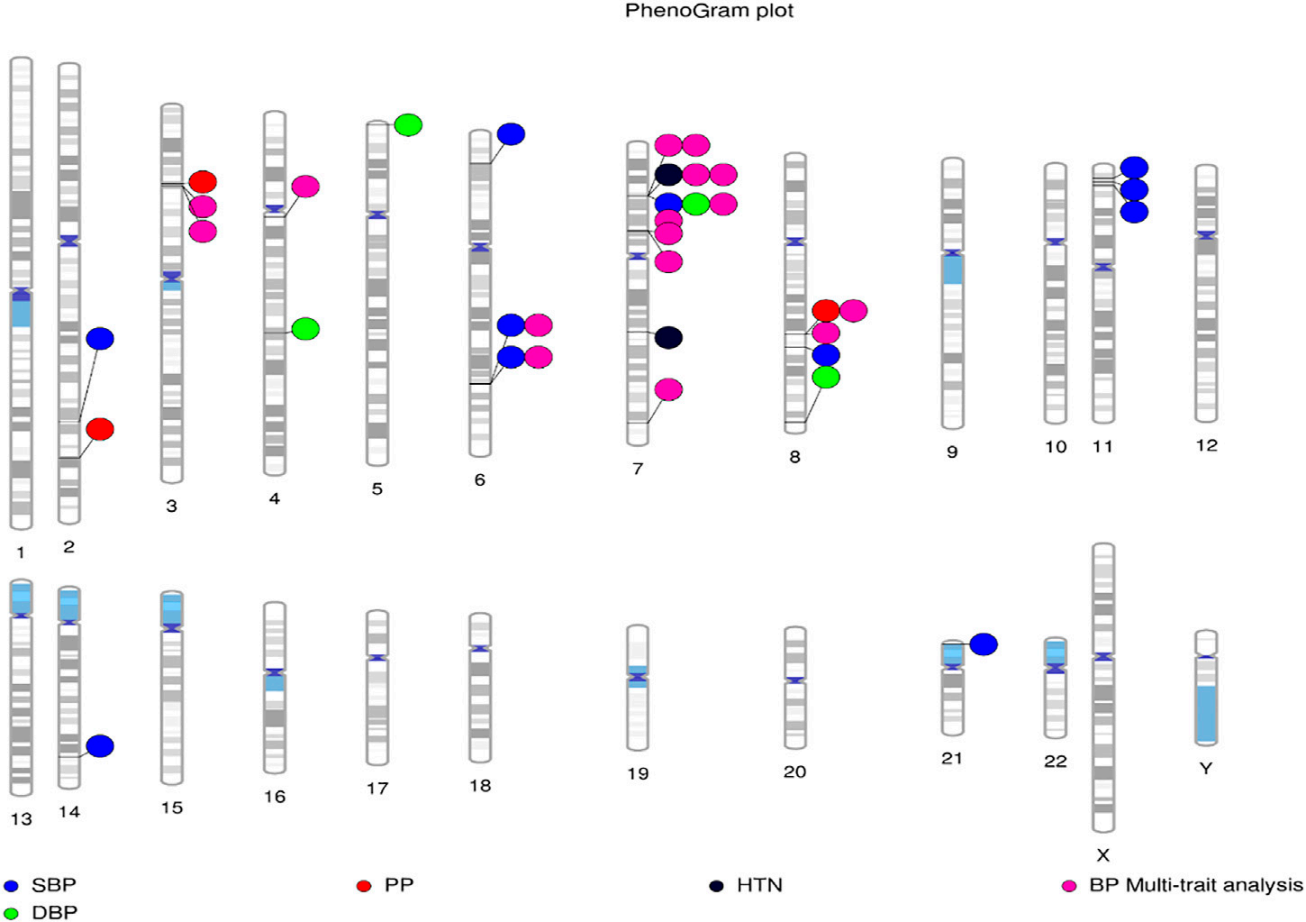
Distribution of loci identified by GWAS for BP-associated traits in African-ancestry populations. Lines are plotted on the chromosomes corresponding to the base-pair location of each SNP, and the coloured circles represent the associated phenotype(s). Constructed using PhenoGram (Wolfe et al., 2013).

**FIGURE 3:**
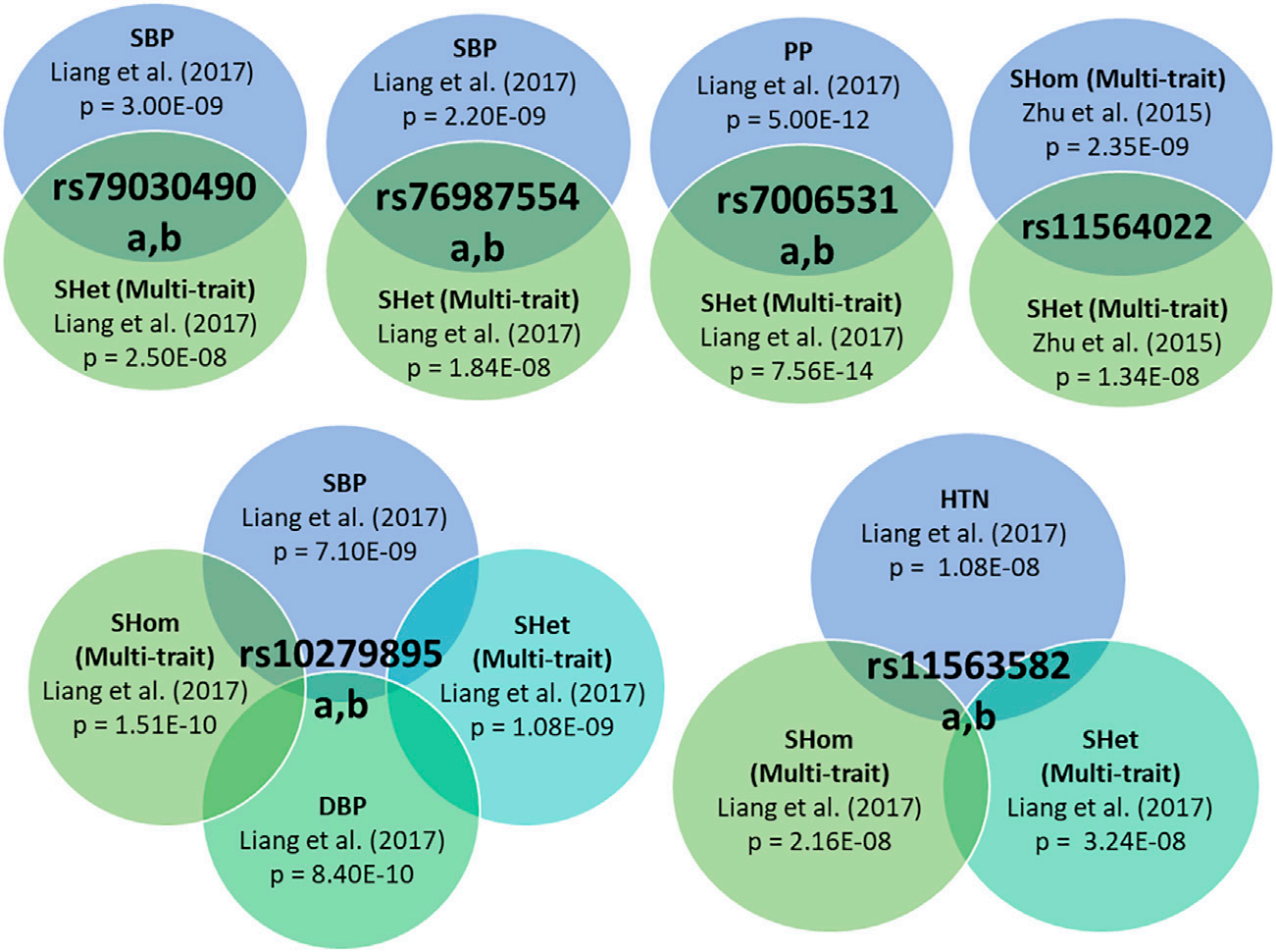
Individual variants with associations to multiple BP-related traits, exhibiting pleiotropic effects.

The analysis of genetic associations was based on the 10 studies with only African-ancestry participants (Table 4). Liang et al. (2017), based on 31968 African-ancestry individuals (largest), reported the most associations across all BP-related traits (SBP, DBP, HTN, PP and BP multi-trait analysis). The distribution of genome-wide signals across the 22 chromosomes, per trait, is shown in Figure 2. The 26 genome-wide significant SNPs found across BP-related traits, are summarized in Table 5 (suggestive associations are shown in Supplementary Table S6). Six SNPs were associated with more than one BP phenotype from the same study (Figure 3), but none of associations were found in more than one study.

1) Genetic associations detected in each study

**TABLE 4:** GWAS associated SNPs detected for BP-related traits in the 10 studies, with 100% African-ancestry participants.

**BP traits**	**BP trait type**	**GW association number**	**Suggestive association number**	**Total associations**	References
SBP	SBP	5	78	83	Adeyemo et al. (2009)
SBP	SBP	1	9	10	Fox et al. (2011)
SBP	SBP	1	18	19	Franceschini et al. (2013)
SBP	SBP (GERA AA)	0	10	10	Hoffmann et al. (2017)
SBP	SBP	4	6	10	Liang et al. (2017)
SBP	SBP	0	4	4	Lule et al. (2019)
SBP	SBP	1	0	1	Zhu et al. (2015)
DBP	DBP	0	82	82	Adeyemo et al. (2009)
DBP	DBP	1	3	4	Fox et al. (2011)
DBP	DBP	0	21	21	Franceschini et al. (2013)
DBP	DBP (GERA AA)	0	6	6	Hoffmann et al. (2017)
DBP	DBP	3	14	17	Liang et al. (2017)
DBP	DBP	0	4	4	Lule et al. (2019)
PP	PP (GERA AA)	0	10	10	Hoffmann et al. (2017)
PP	PP	3	16	19	Liang et al. (2017)
HTN	HTN	0	77	77	Adeyemo et al. (2009)
HTN	HTN	0	4	4	Franceschini et al. (2013)
HTN	HTN	1	0	1	Lettre et al. (2011)
HTN	HTN	1	6	7	Liang et al. (2017)
HTN	HTN	0	1	1	Zhu et al. (2015)
BP multi-trait	BP traits (SHet)	9	17	26	Liang et al. (2017)
BP multi-trait	BP traits (SHom)	2	15	17	Liang et al. (2017)
BP multi-trait	BP traits (SHet)	4	6	10	Zhu et al. (2015)
BP multi-trait	BP traits (SHom)	1	2	3	Zhu et al. (2015)

**TABLE 5:** SNPs associations in the studies with only African-ancestry participants.

**Trait**	**Chr**	**SNP**	**Position**	**Reported Gene**	**Distance to gene (kb)**	**Type**	**EA**	**RA**	**EAF**	**OR or BETA**	**SE or 95% CI**	**Heterogeneity**	*p* **-value**	**Top SNP rank**	**Analysis type**	**Reported Build**	**Ref**
SBP	2	rs5743185	190446083	*PMS1*	0	Intronic	T	NA	0.14	-	-	—	2.09E-11	1	—	GRCh36	Adeyemo et al. (2009)
SBP	6	rs16877320	16031005	*AL365265.23*	-11987	Intergenic	G	T	0.13	-	-	—	3.42E-09	2	—	GRCh36	Adeyemo et al. (2009)
SBP	6	**rs76987554a,b**	134080855	—	—	—	t	c	0.1	−1.85	0.31	—	2.20E-09	1	—	GRCh37	Liang et al. (2017)
SBP	6	**rs79030490a,b**	134087689	—	—	—	a	c	0.1	−1.83	0.31	—	3.00E-09	2	—	GRCh37	Liang et al. (2017)
SBP	7	**rs11563582a,b**	27351650	—	—	—	a	g	0.13	1.61	0.28	—	7.10E-09	3	—	GRCh37	Liang et al. (2017)
SBP	8	rs17365948	102026053	*YWHAZ*	0	Intronic	A	C	0.11	—	—	—	1.59E-08	4	—	GRCh36	Adeyemo et al. (2009)
SBP	11	rs11041530	7658079	NA	—	Intergenic	c	g	0.11	−1.35	0.25	3.45E-01	4.04E-08	1	—	GRCh36	Franceschini et al. (2013)
SBP	11	rs12279202	9388666	*IPO7*	0	Intronic	A	C	0.12	—	—	—	4.80E-08	5	—	GRCh36	Adeyemo et al. (2009)
SBP	11	rs7941684a	5532222	—	—	—	t	g	1.0	−1.23	0.22	—	2.40E-08	4	—	GRCh37	Liang et al. (2017)
SBP	14	rs11160059	91877083	*SLC24A4*	0	Intronic	A	NA	0.28	—	—	—	1.54E-08	3	—	GRCh36	Adeyemo et al. (2009)
SBP	21	rs2258119	18 89 350	*C21orf91*	—	Genotyped	C	T	0.32	1.84	0.34	0.7	4.69E-08	1	—	GRCh36	Fox et al. (2011)
DBP	4	rs62312401a	116987529	—	—	—	a	g	0.94	1.31	0.24	-	3.50E-08	3	—	GRCh37	Liang et al. (2017)
DBP	5	rs10474346	9,05,99,895	*GPR98/ARRDC3*		Imputed	C	T	0.33	1.1	0.2	0.11	3.56E-08	1	—	GRCh36	Fox et al. (2011)
DBP	7	**rs11563582a,b**	27351650	—	—	—	a	g	0.13	1.02	0.17	-	8.40E-10	1	—	GRCh37	Liang et al. (2017)
DBP	8	rs78192203a	142375073	—	—	—	a	t	0.2	−0.77	0.14	-	1.30E-08	2	—	GRCh37	Liang et al. (2017)
PP	2	rs556271823	210033781	—	—	—	a	at	0.1	−1.62	0.29	-	3.10E-08	3	—	GRCh37	Liang et al. (2017)
PP	3	rs114821199a	40965875	—	—	—	a	g	0.01	4.21	0.73	-	1.00E-08	2	—	GRCh37	Liang et al. (2017)
PP	8	**rs7006531a,b**	95110744	—	—	—	a	g	1.0	−1.16	0.17	-	5.00E-12	1	—	GRCh37	Liang et al. (2017)
HTN	7	**rs10279895a,b**	27328210	—	—	—	a	g	1.0	0.19	0.03	-	1.80E-08	1	—	GRCh37	Liang et al. (2017)
HTN	7	rs7801190	100296029	*SLC12A9*	—	—	-	C	0.73	1.35	1.22–1.50	-	2.50E-08	1	—	GRCh36	Lettre et al. (2011)
BP Multi-trait analysis	3	rs147428270b	41868721	—	—	—	t	c	0.63	—	—	—	2.49E-08	6	SHet	GRCh37	Liang et al. (2017)
BP Multi-trait analysis	3	rs7651190b	41765955	—	—	—	a	g	0.4	—	—	—	6.87E-09	4	SHet	GRCh37	Liang et al. (2017)
BP Multi-trait analysis	4	rs11725861	54497062	*CHIC2*	—	—	A	-	0.84	—	—	—	8.45E-09	2	SHet	GRCh36	Zhu et al. (2015)
BP Multi-trait analysis	6	**rs76987554a,b**	134080855	—	—	—	t	c	0.1	—	—	—	1.84E-08	5	SHet	GRCh37	Liang et al. (2017)
BP Multi-trait analysis	6	**rs79030490a,b**	134087689	—	—	—	a	c	0.1	—	—	—	2.50E-08	7	SHet	GRCh37	Liang et al. (2017)
BP Multi-trait analysis	7	**rs10279895a,b**	27328210	—	—	—	a	g	1.0	—	—	—	3.24E-08	8	SHet	GRCh37	Liang et al. (2017)
BP Multi-trait analysis	7	**rs10279895a,b**	27328210	—	—	—	a	g	1.0	—	—	—	2.16E-08	2	SHom	GRCh37	Liang et al. (2017)
BP Multi-trait analysis	7	rs115476423b	149199964	—	—	—	c	g	0.94	—	—	—	4.99E-08	9	SHet	GRCh37	Liang et al. (2017)
BP Multi-trait analysis	7	**rs11563582a,b**	27351650	—	—	—	a	g	0.13	—	—	—	1.08E-09	2	SHet	GRCh37	Liang et al. (2017)
BP Multi-trait analysis	7	**rs11563582a,b**	27351650	—	—	—	a	g	0.13	—	—	—	1.51E-10	1	SHom	GRCh37	Liang et al. (2017)
BP Multi-trait analysis	7	**rs11564022**	27303571	*HOXA-EVX1*	—	—	T	-	0.23	—	—	—	1.34E-08	3	SHet	GRCh36	Zhu et al. (2015)
BP Multi-trait analysis	7	**rs11564022**	27303571	*HOXA-EVX1*	—	—	T	-	0.23	—	—	—	2.35E-09	1	SHom	GRCh36	Zhu et al. (2015)
BP Multi-trait analysis	7	**rs11977526**	45974635	*IGFBP1,IGFBP3*	—	—	A	-	0.32	—	—	—	1.87E-08	4	SHet	GRCh36	Zhu et al. (2015)
BP Multi-trait analysis	7	**rs11977526b**	46008110	-	—	—	a	g	0.34	—	—	—	4.53E-09	3	SHet	GRCh37	Liang et al. (2017)
BP Multi-trait analysis	8	rs2446849	95172673	*CDH17*	—	—	T	-	1.0	—	—	—	7.01E-09	1	SHet	GRCh36	Zhu et al. (2015)
BP Multi-trait analysis	8	**rs7006531a,b**	95110744	-	—	—	a	g	1.0	—	—	—	7.56E-14	1	SHet	GRCh37	Liang et al. (2017)

Ranked by chromosome number for: (a) SBP. (b) DBP. (c) PP. (d) HTN. (e) BP Multi-trait analyses. Consisting of only GW associations (p < 5E-8). Duplicate SNPs in bold. Chr: Chromosome, EA: Effect allele, EAF: Effect allele frequency.

Genome-wide associations were reported for six studies, briefly summarised below.

Adeyemo et al. (2009) reported genome-wide significant associations with SBP for one intergenic SNP (rs16877320 in *AL365265.23*), and four intronic SNPs (rs5743185 in *PMS1*, rs17365948 in *YWHAZ*, rs12279202 in *IP O 7* and rs111600591 in *SLC24A4*). Replication was found in each gene for at least one SNP with low *p*-values for the Diabetes Genetics Initiative study (Saxena et al., 2007). A non-synonymous-coding SNP in *CACNA1H* was also discussed in study, however this SNP just missed the significance threshold (*p* = 6.71E-08) (Supplementary Table S6). Two of these genes (*CACNA1H* and *SLC24A4*), have annotations that suggest a role in BP regulation (Adeyemo et al., 2009).

Fox et al. (2011) identified two novel loci i.e., rs2258119 (genotyped SNP in *C21orf91*) and rs10474346 (imputed in *GPR98/ARRDC3*) for SBP and DBP respectively. These SNPs did not replicate in the AA population. The SNP rs10474346 is within the region of arrestin C, which is a peroxisome proliferator-activated receptor gamma that may play a role in HTN through modification of inflammation and the innate immunity system in vascular cells. A genotyped SNP (rs1990151) in *IP O 7*, which showed suggestive significance with *p* = 7.39E-07 (Table S 6), is within the same importin beta protein gene (*IP O 7*) and may be a potential link between early-onset glucocorticoid exposure and HTN, through changes in gene expression and kidney function (Fox et al., 2011).

Franceschini et al. (2013) found only one genome-wide associated SNP for SBP i.e., rs11041530 which is 10 kb downstream of the cytochrome b5 reductase 2 gene (*CYB5R2 [MIM608342]*). In addition, an intergenic SNP rs17428471 (in *HOXA3*), which showed suggestive significance (p = 4E-07) (Supplementary Table S6), replicated for SBP in additional AA samples (another associated SNP, rs11564022, is also intergenic in *HOXA3*), and was found to be a *trans*-ethic SNP since it was found significant for both SBP and DBP in *trans*-ethnic meta analyses (Franceschini et al., 2013).

Lettre et al. (2011) found one novel genome-wide associated SNP for HTN (rs7801190, in *SLC12A9*; *p* = 2.5E-08). *SLC12A9* is a potassium/chloride transporter gene. However, this SNP was identified using imputed genotypes (imputation quality r2_hat = 0.70) and replication (also obtained by imputation) was not significant (*p* = 0.29), suggesting that this association may be due to chance (Lettre et al., 2011).

Liang et al. (2017) is to date the largest African-ancestry GWAS conducted (*n* = 31968). This study reported 14 independent genome-wide associated SNPs (Table 5). These were spread in 11 independent SNPs at 11 loci, including three novel loci (*TARID/TCF21, FRMD3,* and *LLPH/TMBIM4*). This study also pointed out that the African-specific SNPs were not well tagged by HAPMAP2 data (https://medicagohapmap2.org/) which could have limited the association detection in their previous study i.e., Franceschini et al. (2013). The SNPs identified in this study were found in immune, kidney, heart, and vascular system pathways and suggests that several associated genes may be involved in the kidneys renin-angiotensin pathways kidney during HTN (Liang et al., 2017).

Zhu et al. (2015) detected five genome-wide significant loci for the BP multi-trait analyses i.e., SHom detected the *HOXA-EVX1* locus, whereas SHet detected the loci *CHIC2, HOXA-EVX1, IGFBP1/IGFBP3*, and *CDH17* (Table 5). Three of the SHet associated loci (*CHIC2, HOXA-EVX1, and IGFBP1/IGFBP3*) were confirmed to be associated with HTN-related traits. It was suggested that SHet was more powerful compared to combined *p*-values when accounting for heterogeneity (Zhu et al., 2015).

2) Comparison of genetic associations detected

Six SNPs were pleiotropic (associated with more than one trait) (Figure 3). Liang et al. (2017) identified significant associations with five SNPs of the same polymorphisms for different traits, with most occurring with at least one BP Multi-trait analysis method. Zhu et al. (2015) reported that rs11564022 (HOXA-EVX1) was significant for both BP multi-trait analysis methods (SHet and SHom). This SNP was also found to be suggestively significant in Franceschini et al. (2013) for SBP (*p* = 1.83E-06), DBP (*p* = 7.66E-08) and HTN (*p* = 6.78E-08) (Supplementary Table S6).

The lookup of the 26 genome-wide associated lead SNPs detected in African populations (Table 5), in summary statistics of the largest GWAS to date for BP (Evangelou et al., 2018), found only 14 of 26 SNPs to be present in the study (for SBP, DBP and PP). Of these, 4 SNPs show at least a suggestive *p*-value (rs7651190b, rs11725861, rs11563582a,b, rs11977526b) in this European ancestry based GWAS (Supplementarty Table S7) and the other ten had *p*-values ranging from 1.5E-04 to 9.74E-01.

Furthermore, the look up of these 26 SNPs in other BP related GWASs (based on the GWAS Catalog, with a cut-off value p < 5E-6), detected three SNPs (also found in Evangelou et al. (2018), with p < 5E-6) that were associated with BP in four non-African-ancestry based GWASs. All three SNPs were associated in the African-ancestry population with multiple BP traits using the SHet method: rs7651190b (Liang et al., 2017), was found to be associated with PP in Japanese ancestry based GWAS (Takeuchi et al., 2018). rs11725861 (Zhu et al., 2015), was found to be associated with PP in European ancestry based GWAS (Hoffmann et al., 2017). rs11977526 (Zhu et al., 2015; Liang et al., 2017), was found to be associated with SBP in European ancestry based GWAS (Surendran et al., 2016), DBP in another European ancestry based GWAS (Hoffmann et al., 2017) and PP in both European (Hoffmann et al., 2017) and Japanese (Takeuchi et al., 2018) ancestry based GWAS.

After comparing the 26 genome-wide associated SNPs (Table 5) to the GWAS catalog (with a cut-off value p < 5E-6) and Evangelou et al. (2018) summary statistics, only 12 of the SNPs were observed in studies with African-ancestry participants (Table 6).

**TABLE 6:** Potential unique African-ancestry GW associations signals for 10 studies, based on 100% African-ancestry.

**No**	**Chr**	**SNP**	**Position **	**Reported Gene**	**Distance to gene (kb)**	**Type**	**EA**	**RA**	**EAF**	**Associated Traits**	**Reported Build**	**EuA**	**African-ancestry**	**Higher freq**
1	2	rs556271823	210033781	—	—	—	a	at	0.06	PP	GRCh37	0.1214	0.0483	EuA
2	2	rs5743185	190446083	*PMS1*	0	Intronic	T	NA	0.1418	SBP	GRCh36	0.0696	0.0182	EuA
3	3	rs114821199a	40965875	—	—	—	a	g	0.01	PP	GRCh37	0.0002	0.0145	African-ancestry
4	3	rs147428270b	41868721	—	—	—	t	c	0.63	BP Multi-trait analysis	GRCh37	0.0759	0.6448	African-ancestry
5	6	rs76987554a,b	134080855	—	—	—	t	c	0.09	SBP; BP Multi-trait analysis	GRCh37	0.0003	0.0910	African-ancestry
6	6	rs79030490a,b	134087689	—	—	—	a	c	0.09	SBP; BP Multi-trait analysis	GRCh37	0.0003	0.0931	African-ancestry
7	7	rs10279895a,b	27328210	—	—	—	a	g	0.90	HTN; BP Multi-trait analysis	GRCh37	0.0001	0.09283	African-ancestry
8	7	rs115476423b	149199964	—	—	—	c	g	0.94	BP Multi-trait analysis	GRCh37	6.485E-05	0.0544	African-ancestry
9	8	rs2446849	95172673	*CDH17*	—	—	T	-	0.80	BP Multi-trait analysis	GRCh36	0.9581	0.8059	EuA
10	8	rs7006531a,b	95110744	—	—	—	a	g	0.85	PP; BP Multi-trait analysis	GRCh37	0.0006	0.1497	African-ancestry
11	8	rs78192203a	142375073	—	—	—	a	t	0.20	DBP	GRCh37	0.0003	0.1961	African-ancestry
12	14	rs11160059	91877083	*SLC24A4*	0	Intronic	A	NA	0.1782	SBP	GRCh36	1.0000	0.9288	EuA

b Ranked by chromosome number for: (a) SBP. (b) DBP. (c) PP. (d) HTN. (e) BP Multi-trait analyses. Consisting of only GW associations (p < 5E-8). Chr: Chromosome, EA: Effect allele, EAF: Effect allele frequency, EuA: European allele frequency (Average of Finnish and non-Finnish population).

## Discussion

This systematic review of genetic association studies for BP, HTN and related phenotypes in African-ancestry populations, has highlighted the need for further studies from Africa, where HTN is most prevalent.

Despite the first GWAS with BP and HTN being conducted in 2007 and there being over 160 published studies, there are just a handful of studies on African-ancestry participants and even fewer on continental African populations. It is however encouraging that the number of studies is increasing and that sample sizes are also becoming larger in individual studies, although many remain small (e.g., Willems et al. (2020) had only 281 African-ancestry participants). Large sample sizes, especially in continental African populations are necessary to enhance our understanding of the genetic architecture of BP traits.

Meta-analysis of studies from diverse populations can lead to higher power for detecting associations across multiple populations and can begin to address the transferability of associations across populations (Willems et al., 2020).

### Summary of Studies and Associations

The variability between associations found across studies may be partly attributable to differences in study protocols between studies, but could also be due to major differences in sample size and sampling geography. Regional/geographical variability may also play a major role in the differences observed in genetic architecture. Most studies reported mainly on AA participants (Table 1), with African studies limited to Nigeria (Franceschini et al., 2013; Zhu et al., 2015) and Uganda (Gurdasani et al., 2019; Lule et al., 2019), and the origin of eight specified “African-ancestry” studies were not mentioned. African American populations are admixed and according to several studies and the recorded history of the trans-Atlantic slave trade, predominately represent a West African-ancestry, and is limited to specific countries or regions (Tishkoff et al., 2009; Patin et al., 2017). Therefore, genetic associations may differ within groups of AAs due to regional variability in the origins of their African ancestors, with a potential genetic stratification bias which would also be affected by the proportion of European ancestry in different participants and groups (Zakharia et al., 2009). Studies focusing on a selection of other countries within Africa are necessary to capture the ethnic diversity across sub-Saharan Africa and the role of genetic variation in BP related traits (Ramsay et al., 2016; Choudhury et al., 2020).

For studies that provided details on how phenotype data was collected, there was variability in the instrument used and how readings were taken to measure SBP and DBP, and the guidelines used to determine HTN and adjustments made for AHM (Table 2). Variability of BP-related phenotype traits in different populations could influence genetic associations, and may be partly attributed to environmental factors and comorbidities, as well as different age ranges in the participants.

The use of AHM was reported by four studies (Fox et al., 2011; Franceschini et al., 2013; Liang et al., 2017; Giri et al., 2019), however, the methods used for adjustments were not always mentioned. This may introduce bias when making comparisons across studies, as studies that make adjustments would not be comparable to studies that did not make adjustments. Two studies conducted BP multi-trait analyses (Zhu et al., 2015; Liang et al., 2017), using the SHet and SHom approaches. It was reported that these methods gave greater power to detect associations and the SHet method resulted in more associations compared to SHom for both studies. SHet was able to capture evidence for association, even when there was noise caused by heterogeneity among traits and cohorts (Zhu et al., 2015).

Data and statistical models were different in terms of final array sizes (depending on the genotyping array, imputation strategy and reference panel used), GWAS method (depending on adjustments for population structure/PCA (principal components analysis), association model, covariates adjusted for) and thresholds used for determining suggestive significance (Table 3). Adjusting for age, age2, sex and the first 10 PCs, is common practice for most GWASs, however, the reason BMI is used as an adjustment variable for most studies is not mentioned in the studies. This may have to do with the strong associations found between BMI and high BP/HTN (Landi et al., 2018).

### Lack of Power to Detect Small Effects

The lack of significant associations found for BP traits from several of the African-ancestry studies is largely due to the low power to detect small effects of common SNPs, and the fact that genotyping arrays are usually Eurocentric and enriched for common European SNPs. Given that sample sizes tend to be smaller in African-ancestry studies, they only have power to detect large effect associations. Therefore, many true associations with small effects will not be significant and many suggestive associations may be spurious. The use of multiple cohorts, to maximize sample size and increase power, could bias findings as some associations may be due to heterogeneity in BP measurements across the different cohorts (Fox et al., 2011).

Many genetic associations found in European studies may not replicate because of the low power of the small studies to detect small effect variants. It is well recognised that BP traits are influenced by many genetic variants, with each conferring a small change in BP, and modulated by environmental factors, behaviour and other physiological markers (Lule et al., 2019). This leads to failure to replicate most associated variants, since many GWAS are only powered to identify common variants of moderate to large effects (Adeyemo et al., 2009; Lettre et al., 2011). Since the African-ancestry samples are from different geographical regions and data is so limited, population admixture may result in different linkage disequilibrium (LD) patterns across African populations, thus limiting replication across different African populations (Fox et al., 2011). Failure to replicate SNPs in Lule et al. (2019) could be due to differences in MAF across the different populations or differences in LD patterns, as well the lack of knowledge of causative variants or due to spurious initial findings.

### The Importance of Africa-Centric Arrays, Panels and Africa-Ancestry Studies

GWAS arrays are designed to capture common variation and rely on LD with causative variants to detect association. Most SNP arrays have generally been enriched for common European variants, suggesting that there may be many common African-ancestry SNPs that have not yet been identified as being associated with specific traits (Hoffmann et al., 2017). Interestingly, 14 of the 26 genome-wide SNPs associated in African-ancestry studies were also found in Evangelou et al. (2018) and 12 were potentially unique to African-ancestry populations (Table 6). This could, in part, be due to differences in linkage disequilibrium, genomic coverage of SNPs and allele frequencies that could lead to differences in associations between African and non-African-ancestry groups. African populations have over half a million more SNPs sites per genome in comparison to Europeans, therefore, GWASs performed on this population require more SNPs with a wide genomic coverage to ensure that most LD blocks are covered in the analyses (Ramsay, 2012; Choudhury et al., 2020). The development of Africa-centric arrays are important to detect signals that are unique to African-ancestries. One example of such an array is the H3Africa SNP array, consisting of ∼2.3 million SNPs, including a large proportion of common African variants that effectively tag haplotype blocks in relevant populations and also included disease relevant variants from databases (such as the GWAS Catalog, ClinVar, and Cosmic).

African specific imputation panels are also important to better capture African specific variants. The AGVP reference panel, which includes 320 whole-genome sequences from 320 individuals across sub-Saharan Africa, has an efficient genotype array design capturing common genetic variation in Africa (Gurdasani et al., 2015) and hence this panel was used by both studies Ugandan studies. Moreover, the availability of larger panels, such as the TopMed with substantial African-ancestry representation, is expected to enhance the quality of imputation and thereby increasing the number of variants that could be included in future association studies (Bentley et al., 2020). This might also benefit the meta-analysis with some of the existing GWAS data.

This review emphasizes the value of including African-ancestry studies, as there are BP and HTN signals unique to African populations. Therefore, further studies in this under-represented region are needed to identify more associations and to validate signals that appear to be unique to African-ancestry populations. Since most GWAS that include African-ancestry participants are AA, the results reported here are unlikely to be widely transferable to populations in Africa and emphasize the need for future studies based in continental Africa.

### Limitations

There is a limited body of literature on GWAS in populations of African-ancestry, mainly represented by AA. This is well recognized in summaries of GWAS findings and the universal call for more representation of Africans and minority populations in such studies. Variations between population characteristics, phenotypic measures and genotypic analyses could cause bias when comparing different studies. It is therefore important for all studies to report more detail in the protocols to enable better replication and minimise bias between studies. Recent studies suggest that the genetic architecture of BP is different between longitudinal trajectories and cross sectional studies and therefore cohort analyses would be important in African settings. The relatively low overlap between signals detected in African and non-African-ancestry GWASs could be due to LD differences and the same locus being represented by different lead SNPs in the two groups. Although, a regional replication was not performed in the current review, which might have enhanced the overlap, we anticipate that many of the African-specific signals could indeed be continent-specific. Due to these limitations, the results are essentially a list of sentinel findings. A narrative synthesis of the results was conducted (limited to reporting lead SNPs), as a meta-analysis was not possible and additional analyses, such as regional mining or looking at credible sets, were challenging.

### Future Directions

Genetic diversity is high within African populations, because of a deep evolutionary history, population admixture and genetic drift. It is therefore vital for future studies to capture contributions from African studies and to understand the functional and biological relevance of associated SNPs. Improved methods need to be developed to understand and compare heritability across populations and studying participants from other parts of the African continent, such as South and Central Africa are essential.

For enhanced discovery in GWAS in Africans, future studies should aim for increased sample size, multiple BP-related traits (including SBP, DBP, HTN, PP, MAP, BP multi-trait analysis and longitudinal data) to enhance discoverability across phenotypes, the use of Africa-centric genotyping arrays and larger imputation reference panels, which adequately represent African-genetic diversity (Hoffmann et al., 2017).

## Conclusions

There is much need for more genetic-association studies based on BP and HTN in large-scale African-ancestry research, specifically in participants from the African continent. Further studies should also investigate rare variants, adjust appropriately for the use of anti-hypertensive medication, follow standardised procedures and provide justification for the different approaches used.

## Data Availability

The original contribution presented in the study is included in the article/Supplementary Material, further inquiries can be directed to the corresponding authors.

## Abbreviations

AA, African-American; AHM, antihypertensive medication; AWI-Gen Africa Wits-INDEPTH Partnership for Genomic Research; BMI, body mass index; BP, blood pressure; CARe, candidate gene association resource; COGENT, continental origins and genetic epidemiology network; DBP, diastolic blood pressure; pressure in the arteries when the heart rests between beats, measured as a continuous trait; GW, genome-wide; GWAS, genome-wide association study; HTN, hypertension; JNC7, guidelines were used i.e., HTN if SBP ≥140 mmHg and/or DBP>= 90 mmHg and/or treatment with AHM, measured as a binary trait; LD, linkage disequilibrium; LMM, linear mixed model; LoRM, logistic regression model; LRM, linear regression model; MAF, minor allele frequency; MAP, mean arterial pressure; sum of DBP and a third of the PP (i.e., DBP +1/3 PP), measured as a continuous trait; PC, principle component; PCA, principle components analysis; PP, pulse pressure; difference between the SBP and DBP (i.e., SBP-DBP), measured as a continuous trait; SBP, systolic blood pressure; pressure in the arteries when the heart beats, measured as continuous trait; SHet extension of SHom and it can increase the statistical power over SHom when a variant affects only a subset of traits; The distribution of SHet under the null hypothesis is obtained through an estimated beta distribution; SHom, Similar to fixed effect meta-analysis method but accounts for the correlation of summary statistics of the multi-traits and for overlapping or related samples among the cohorts; Uses the trait sample size as the weight, so that it is possible to combine traits with different measurement scales; SNP, single nucleotide polymorphism.
