# Supplementary material for: Systematic Review of Genomic Associations with Blood Pressure and Hypertension in Populations with African-Ancestry
Source: Front Genet. 2021 Oct 20;12:699445. doi: 10.3389/fgene.2021.699445 (PMC8564494; doi:10.3389/fgene.2021.699445)
Supplement: Supplementary file 1 [file Image1.pdf]

Supplementary Material

1 Supplementary Figures

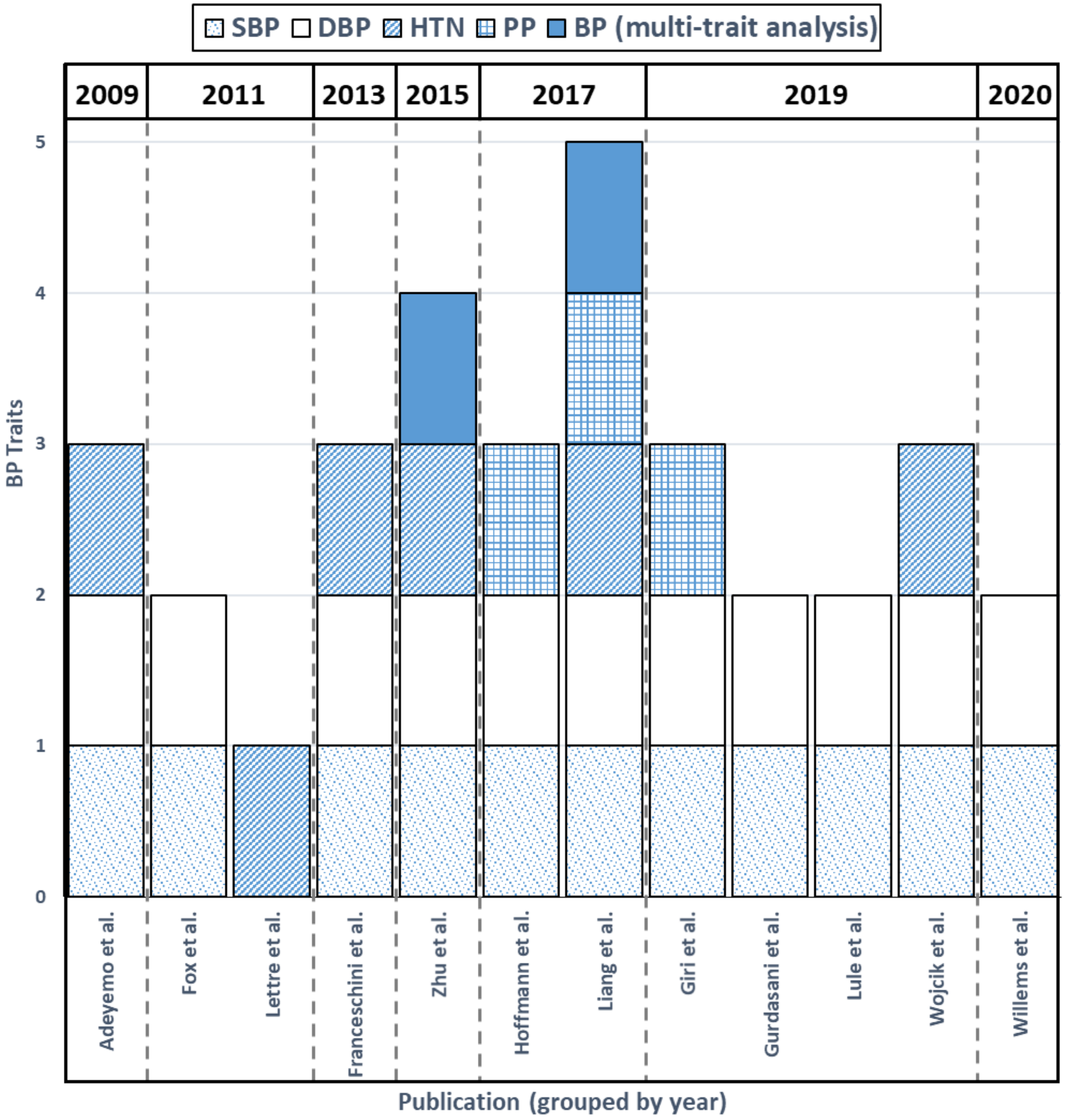

Figure S 1: Distribution of BP traits for the 12 eligible publications (by year)
